# Violence, Harassment, and Turnover Intention in Home and Community Care: The Role of Training

**DOI:** 10.3390/healthcare11010103

**Published:** 2022-12-29

**Authors:** Firat K. Sayin, Margaret Denton, Catherine Brookman, Sharon Davies, Isik U. Zeytinoglu

**Affiliations:** 1 Department of Management, Sobey School of Business, Saint Mary’s University, Halifax, NS B3H 3C3, Canada; 2 Department of Health, Aging & Society, McMaster University, Hamilton, ON L8S 4L8, Canada; 3 Catherine Brookman Consulting & Associates, Hamilton, ON L8S 4L8, Canada; 4 DeGroote School of Business, McMaster University, Hamilton, ON L8S 4L8, Canada; 5 Human Resources and Management Area, DeGroote School of Business, McMaster University, Hamilton, ON L8S 4L8, Canada

**Keywords:** intention to stay, workplace violence and harassment, conservation of resources theory, job demands–resources theory, workplace training, stress, self-esteem, personal support workers, health support workers, home care organizations, community care organizations

## Abstract

Background: Violence and harassment affect healthcare workers’ well-being and career decisions in the home and community care sector. Purpose: The objective of this study is to assess the role of training in alleviating the relationship between violence and harassment at work and turnover intention among personal support workers (PSWs). Methodology/Approach: Cross-sectional survey data from 1401 PSWs in Ontario, Canada are analyzed with structural equation modeling. Utilizing a resource perspective, the associations between job demands (i.e., violence and harassment at work), personal resources (i.e., self-esteem), job resources (i.e., workplace violence training and challenging task training), stress, and intention to stay among personal support workers (PSWs) are examined. Results: Challenging task training is positively associated with self-esteem and negatively associated with stress, whereas workplace violence training does not have a significant association with either variable. Stress has a negative relationship with intention to stay. Self-esteem is the mediator of both associations between violence and harassment at work and stress and between challenging task training and stress. Discussion: The results point to varied degrees of training effectiveness that may be shaping turnover decisions of PSWs who experience violence and harassment in home and community care organizations. Practice implications: There seems to be a need to assess and redesign workplace violence training. Home and community care managers might be able to lower the impact of violence and harassment on PSWs’ turnover by providing training that is not directly related to workplace violence and harassment.

## 1. Introduction

Violence and harassment at work is a serious concern in the healthcare sector in Canada and elsewhere. A 2019 report prepared for the House of Commons Canada states that healthcare workers are exposed to workplace violence significantly more than any other profession in Canada [1]. Thus, violence and harassment are increasingly recognized as critical determinants of healthcare workers’ well-being and career decisions [2,3]. This paper examines the relationship between personal support workers’ (PSWs) job demands that result in violence and harassment at work, personal resource of self-esteem, job resources of workplace violence training and challenging task training, PSWs’ well-being of stress, career decisions and organizational outcome of intention to stay in the organization. The analysis utilizes a resource perspective, bringing together job demands and resources theory [4] and conservation of resources theory [5]. 

Violence and harassment in healthcare workplaces can lead to detrimental worker outcomes related to stress [3] and organizational outcomes of turnover intentions [2]. Training is a job resource used to prevent or alleviate the negative outcomes of violence and harassment in the healthcare sector [6]. Research on workplace violence training in the healthcare sector, however, shows inconclusive results. For example, a study conducted in community care organizations found that there was no difference in workplace violence and harassment exposure among those who received workplace violence training and those who did not [7]. On the other hand, Nachreiner et al. [8] demonstrated that workplace training increased the probability of exposure to workplace violence. Understanding the effectiveness of specialized training related to violence and harassment in the healthcare sector is imperative in reducing stress and retaining PSWs. 

While training as a job resource is important in shaping PSWs’ experience of violence and harassment at work, the resource perspective argues that violence and harassment at work as work stressors can be ameliorated with the interplay of the personal resource of self-esteem and the job resource of training. Specifically, we examine the buffering roles of self-esteem as a personal resource and workplace violence training and challenging task training as job resources in the relationship between PSWs’ exposure to violence and harassment at work, stress, and intention to stay. Furthermore, we investigate the reciprocal relationship among self-esteem, workplace violence training, and challenging task training to apprehend how different resources may be associated with each other. Our data comes from 1401 PSWs in Ontario, Canada. 

Our study has important contributions to theory and knowledge. The study contributes to theory by shedding light on the resource perspective. Furthermore, we contribute to practitioner knowledge by examining the effectiveness of two types of training received by PSWs: workplace violence training and challenging task training. It is important to understand the effectiveness of these trainings not only for improving the well-being of PSWs but also for retaining PSWs in their workplaces. 

### 1.1. Background to the Study

There is an increased demand for home and community care services in Canada and most industrialized countries due to demographic shifts and healthcare sector restructuring [9]. PSWs play a key role in addressing the demand for home and community care services. They provide care to individuals such as post-acute patients, older people, and people with disabilities who need support with their everyday tasks to live independently. PSWs are also known in other countries as health support workers, social and healthcare assistants, home healthcare aides, home healthcare workers, and home care workers. PSWs’ workplaces include care recipients’ dwellings, supportive housing programs, and community care, long-term care, and retirement homes. In accordance with the need for their services, PSWs constitute the largest group of providers in the Canadian home and community care sector. 

PSWs, as frontline workers in the home and community care sector, experience violence and harassment at work [10]. Violence and harassment at work can be at least partially avoided by allocating necessary job resources to workers. Thus, human resource policies regarding organizational resources can shape PSWs’ exposure to violence and harassment at work. The topic receives extensive attention by all stakeholders, including the health and safety associations providing health and safety training and resources to healthcare employers, unions, and workers, though the extent of training uptake by workers is not known. 

The PSWs included in this study work in Ontario, Canada. Ontario has occupational health and safety legislation (e.g., Occupational Health and Safety Act, R.S.O. 1990, c. O.1) that designate responsibilities of healthcare organizations to address workplace violence and harassment. Nevertheless, these responsibilities, including training addressed to alleviate the adverse effect of workplace violence and harassment, are vague and left to employer discretion [6]. Thus, training of PSWs is a high-priority issue for the home care sector in Canada [9], especially because it is an important determinant of retention [11]. The challenges of violence and harassment experienced by the PSWs and the interest of their employing organizations, unions, and professional organizations in assisting the workers, along with the provincial government’s interest in documenting this phenomenon, are the impetus for our study.

### 1.2. Theory

The theoretical foundation of this study is based on the resource perspective, which is the integration of the job demands and resources (JD-R) and conservation of resources (COR) theories [12]. According to the JD-R theory, work environment characteristics can be categorized into two groups, as job demands and job resources [4]. Both job demands and resources can be physical, social, psychological, or organizational [13]. The JD-R theory states that job demands lead to strain but job resources can buffer the impact of job demands on strain. Bakker and Demerouti [4] offer an extended model based on the JD-R theory where personal resources are incorporated into the model. In this model, job resources and personal resources interact and act as a buffer between job demands and strain [4]. According to the COR theory, the prime motivation of human beings is to gain, protect, and accumulate resources such as health, well-being, and personal characteristics that may assist in dealing with stress [5,14]. Furthermore, individuals attempt to acquire resources to recover from losses [15] In the workplace context, workers use their personal resources to cope with job demands [16]. Workers may experience stress when job demands exceed personal resources [5]. 

**Figure 1:**
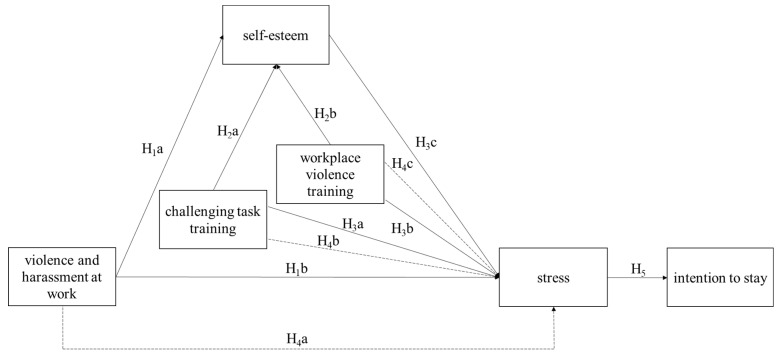
Hypothesized relationships between intention to stay, violence and harassment, challenging task training, workplace violence training, self-esteem, and stress. Notes: Solid lines indicate direct effects; dashes indicate indirect effects.

The model developed in this paper is guided by the organizational health framework developed by Hart and Cooper [17]. Hart and Cooper’s [17] framework argues that organizational and individual characteristics interact to shape employee well-being, which in turn, affects organizational outcomes. We build on this framework using the resource perspective [12] and develop the model of violence and harassment at work, training, self-esteem, stress, and intention to stay relationships for PSWs. The model integrates job demand (violence and harassment at work), job resources (challenging task training and workplace violence training), personal resources (self-esteem), employee well-being (stress), and organizational performance (intention to stay). In applying the JD-R theory to PSWs in our study, we argue that when PSWs experience violence and harassment at work, self-esteem acts as a buffer reducing the effect of violence and harassment at work. The model can be seen in Figure 1. 

Self-esteem is one’s evaluations of self in life [18] and is a personal resource according to the JD-R theory [13,14,15,19]. The JD-R theory states that job demands lead to strain [4], which can then manifest itself as stress. The relationship between home care workers’ exposure to workplace violence and stress was previously documented in Canada [10]. As presented in our model (see Figure 1), we argue that violence and harassment at PSWs’ work increase their level of stress. Thus, we hypothesize that:

**Hypothesis 1a** **(H1a):** *Violence and harassment at work is negatively associated with self-esteem.*

**Hypothesis 1b** **(H1b):** *Violence and harassment at work is positively associated with stress.*

As PSWs are exposed to violence and harassment at work, they face the risk of losing an important personal resource: self-esteem. We argue that challenging task training and workplace violence training, the types of workplace training examined in this paper, can contribute to self-esteem. Challenging task training is essentially a critical thinking skills training that is broad enough to cover a number of challenging circumstances that PSWs might experience at work and provides skills to identify and resolve those challenges. Challenging tasks training can include assisting with the complex healthcare needs of clients, dealing with irritated clients whose needs are not being met, training to manage too many different tasks at the same time while delivering good quality care, and training to care for clients too sick to be at home. Workplace violence training can help to increase PSWs’ awareness of risky situations and prevent problems from happening before they further escalate [20]. Workplace violence training can include recognizing and knowing how to respond to the threat of, attempt to, or exercise of physical strength against the PSW. This may be physical violence (i.e., scratching, pinching, pushing, spitting, slapping/hitting, kicking, biting, punching, restraining) or sexual violence. Workplace violence training can also include recognizing and knowing how to respond to harassing behavior that demeans, humiliates, annoys, alarms, or verbally abuses the PSW or is considered by the PSW as unwelcome. This harassing behavior may be words, gestures, intimidation, bullying, or other inappropriate activities. PSWs who are trained in recognizing workplace violence and harassment and taking measures to prevent the occurrence of violence and harassment have higher self-esteem, with training positively contributing to self-esteem. Thus, training can be an important job resource that enhances PSWs’ personal resource of self-esteem. Therefore, we hypothesize that:

**Hypothesis 2a** **(H2a):** *Challenging task training is positively associated with self-esteem.*

**Hypothesis 2b** **(H2b):** *Workplace violence training is positively associated with self-esteem.*

Stress occurs as a result of interaction between individuals and their environments [5,14]. This implies that job demands, personal resources, and job resources interact to influence stress. According to the JD-R theory, job demands may lead to stress, and job resources and personal resources buffer this relationship [4] The COR theory states that the loss of personal resources such as self-esteem can lead to stress [15]. For example, Yang, Ju, and Lee [21] demonstrated the negative relationship between job stress and self-esteem. Thus, job resources, including challenging task and workplace violence training, are expected to have a negative relationship with stress. Furthermore, the personal resource of self-esteem should have a negative relationship with stress. Therefore, we hypothesize that:

**Hypothesis 3a** **(H3a):** *Challenging task training is negatively associated with stress*.

**Hypothesis 3b** **(H3b):** *Workplace violence training is negatively associated with stress.*

**Hypothesis 3c** **(H3c):** *Self-esteem is negatively associated with stress.*

The JD-R model argues that job resources can act as a buffer between job demands and strains [4]. For example, Xanthopoulou et al. [22] demonstrated the mutual relationship between job and personal resources. However, how job demands, job resources, and personal resources interact to shape strain has not been definitively explained [4]. Demerouti and Bakker [23] suggested that personal resources can be included in the JD-R model as mediator. According to the resource perspective [12], it is possible that personal resources can have a multiple mediator role. Specifically, we argue that the personal resource of self-esteem mediates the relationship between job demands of violence and harassment at work and stress as well as the relationship between the job resources of challenging task training and workplace violence training and stress. As such, we hypothesize that:

**Hypothesis 4a** **(H4a):** *Self-esteem mediates the relationship between violence and harassment at work and stress.*

**Hypothesis 4b** **(H4b):** *Self-esteem mediates the relationship between challenging task training and stress.*

**Hypothesis 4c** **(H4c):** *Self-esteem mediates the relationship between workplace violence training and stress.*

Both job resources and personal characteristics can shape turnover intention in organizations [24]. Intention to stay is a strong indicator of actual turnover behavior [25,26], which is an important outcome considering the potential adverse impact of turnover behavior on organizational outcomes. Previous research showed that stress can lower intention to stay [27]. Furthermore, it was found that stress has a positive relationship with intention to quit [28]. While intention to stay and intention to quit may not be exactly opposite, there is a high level of similarity between them. Thus, we hypothesize that:

**Hypothesis 5** **(H5):** *Stress is negatively associated with intention to stay.*

## 2. Materials and Methods

### 2.1. Research Design and Data Collection

The current study is a part of a larger project aimed at examining occupational health and safety of home- and community-based PSWs in Ontario, Canada. Our project is guided by a research advisory committee, which consisted of representatives from home care organization associations, PSW associations, health and safety associations with an expertise in PSWs, unions, and the project research team (see Acknowledgments section). In this study, we use our 2015 cross-sectional survey. The survey was administered online on our project website. Printed mail-out surveys were also sent to respondents upon request. We encouraged participants to complete the entire survey and provided minor incentives such as gift cards to improve the response rate while underlining the voluntary nature of the survey.

### 2.2. Population and Sample

Our survey respondents are PSWs employed in the home and community care sector in Ontario, Canada. The PSW work is a female-dominated occupation, and similarly, 93.1 percent of our respondents are females. The average age of our respondents is 48.5. Personal support work is not a regulated profession and there is no mandatory registry in Ontario. The number of PSWs working at the home and community care context in Ontario was estimated as 26,000 in 2011 [29]. 1,746 respondents submitted the survey electronically or mailed a completed hard copy of the survey back to us. The sample size of the current study is 1401 after listwise deletion. 

### 2.3. Measures

We used previously published scales in this study. As Table 1 demonstrates, there is a high internal reliability for all scales, ranging from 0.79 to 0.91. Dichotomous variables are coded 1 for agreeing and 0 for disagreeing with the statement. Items are measured on a five-point Likert scale anchored with 5 as ‘strongly agree’ and 1 as ‘strongly disagree’.

#### 2.3.1. Dependent Variable

Intention to stay is a three-item scale by Lyons [30]. An example scale item is ‘You would like to stay at your organization for a long time’.

#### 2.3.2. Independent Variables

Violence and harassment at work, challenging task training, and workplace violence training are dichotomous variables. Violence and harassment at work is measured with ‘In your job as a PSW in the community, in the past 12 months, have you been a victim of physical or sexual violence or harassment at work?’ Challenging task training is measured with ‘Your organization provides you with the appropriate training to handle challenging tasks’. Workplace violence training is measured with ‘What types of health and safety training have you had at your organization? Please choose all that apply: workplace violence training’.

#### 2.3.3. Mediating Variables

Self-esteem and stress are the mediators of this study. We use a global (i.e., general) conceptualization of self-esteem. A global conceptualization of self-esteem has been shown to be related to work conditions such as workplace violence and harassment, job satisfaction, and job rewards [19]. Furthermore, Jex and Elacqua [18] suggest that it can be appropriate to use a global conceptualization of self-esteem if a general stress measure is used. Thus, we use a global conceptualization of self-esteem since we also use a general stress measure. Self-esteem is measured with a six-item scale developed by Pearlin and Schooler [31]. A sample item is “during the past month: on the whole, satisfied with yourself’. The second mediator, stress, is measured with a 14-item symptoms of stress scale by Denton, Zeytinoglu, Davies, and Lian [32]. A sample item is “Below is a list of the ways that some people feel. During the past month: (a) able to sleep through the night (reversed), (b) irritable and tense.”

#### 2.3.4. Control Variables

Tenure and education are the control variables. Rodwell and Demir [33] showed that tenure can affect the exposure of workplace violence among healthcare workers. We measured tenure with years working as a PSW in the home and community care sector. Education is included to account for human capital factors besides tenure and categorized into four dichotomous variables: high school (reference), trade school, college, and university.

### 2.4. Analysis

We used procedural and statistical remedies to alleviate common method bias. We followed procedural remedies such as conducting a pilot study of the survey in both online and print format and balancing positive and negative items as suggested by Podsakoff, MacKenzie, and Podsakoff [34]. As a statistical remedy, we added a first-order factor with all measures and did not find any items with significant factor loadings [35]. We examined multicollinearity by calculating bivariate correlations and variance inflation factors. The strongest bivariate correlation is 0.65, which is lower than the threshold of 0.80 suggested by Meyers et al. [36]. Thus, we believe our analysis does not suffer from multicollinearity.

We used STATA 15 to conduct structural equation modeling (SEM) with maximum-likelihood estimation. We compared the fit indices of full mediation (i.e., no direct effect between violence and stress) and partial mediation (i.e., direct effect between violence and harassment at work) models. The partial mediation model had a better fit than the full mediation model. Thus, we present the partial mediation model as our final model. The goodness-of-fit indices (i.e., root mean square error of approximation, standardized root mean square residual, comparative fit index, Tucker–Lewis index) indicate that our final model has an adequate fit: χ^2^ = 2240.48, d.f. 371; RMSEA = 0.06; SRMR = 0.06; CFI = 0.86; TLI = 0.84.

## 3. Results

### 3.1. Descriptive Statistics

Table 1 indicates that the PSWs in our sample have a moderately high intention to stay, high self-esteem, and moderately low stress. About one-fifth of the respondents experienced violence and harassment at work in the past 12 months prior to data collection. Most PSWs received challenging task or workplace violence training and hold a college degree or above. Average years of work experience as PSWs approximated to 10 years.

### 3.2. Correlations

The bivariate correlations can be seen in Table 1. Intention to stay is negatively correlated with violence and harassment at work and stress and positively correlated with challenging task training, workplace violence training, and self-esteem. Violence and harassment at work has a negative correlation with challenging task training and self-esteem, positive correlation with stress, and a statistically non-significant relationship with workplace violence training. Challenging task training is positively associated with workplace violence training and self-esteem and negatively correlated with stress. Workplace violence training does not have a significant correlation with self-esteem and stress. Finally, stress has a strong negative association with self-esteem.

### 3.3. SEM Analysis

**Figure 2:**
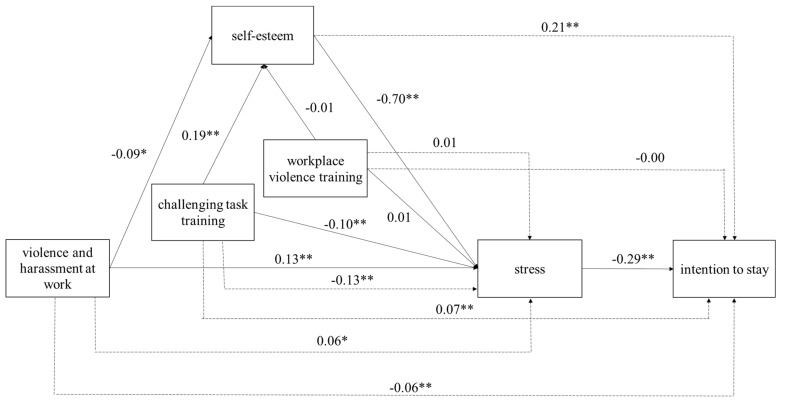
Direct effects between intention to stay, violence and harassment, challenging task training, workplace violence training, self-esteem, and stress. All coefficients are standardized. The control variables are tenure and education (high school or less (reference), trade school, college, university or higher). * *p* < 0.01; ** *p* < 0.001, and *p* > 0.05 for the remaining associations. Notes: N = 1401.

The direct effects can be seen in Figure 2. All coefficients are standardized for ease of interpretation.

Violence and harassment at work is negatively associated with self-esteem (*β* = −0.09, *p* < 0.01) and positively associated with stress (*β* = 0.13, *p* < 0.001), supporting Hypotheses 1a and 1b, respectively. Challenging task training is positively associated with self-esteem (*β* = 0.19, *p* < 0.001); thus, Hypothesis 2a is supported. Workplace violence training does not have a significant association with self-esteem (*β* = −0.01, *p* > 0.05), rejecting Hypothesis 2b. Challenging task training is negatively associated with stress (*β* = −0.10, *p* < 0.001), workplace violence training is not significantly associated with stress (*β* = 0.01, *p* > 0.05), and self-esteem is negatively associated with stress (*β* = −0.70, *p* < 0.001). Thus, Hypotheses 3a and 3c are supported and Hypothesis 3b is rejected.

We employ indirect effects to test mediation relationships (i.e., Hypotheses 4a-c) as suggested by Preacher and Hayes [37]. The indirect effects can be seen in Figure 2. The results indicate that stress has an indirect positive association with violence and harassment at work (*β* = 0.06, *p* < 0.01), negative association with challenging task training (*β* = −0.13, *p* < 0.001), and a non-significant association with workplace violence training (*β* = 0.01, *p* > 0.05). Thus, Hypotheses 4a and 4b are supported and Hypothesis 4c is rejected. These results indicate that self-esteem mediates the associations between violence and harassment at work and stress, and challenging task training and stress, but does not mediate the workplace violence training and stress association. Finally, Hypothesis 5 tests the direct association between stress and intention to stay, and it is supported (*β* = −0.29, *p* < 0.001).

The remaining indirect effects indicate that intention to stay is negatively associated with violence and harassment at work, positively associated with challenging task training and self-esteem, and not significantly associated with workplace violence training. These results are in line with our hypotheses, except the non-significant association between workplace violence training and intention to stay.

Most of the control variables do not have statistically significant associations. Among direct effects, stress is positively associated with college (*β* = 0.08, *p* < 0.05) and university (*β* = 0.08, *p* < 0.01). The only statistically significant indirect effect is the negative association between intention to stay and college (*β* = −0.03, *p* < 0.05).

## 4. Discussion

This study found that violence was related negatively to self-esteem and positively to stress. Self-esteem was positively associated with challenging task training, did not have a significant relationship with workplace violence training, and negatively associated with stress. We showed that stress was not significantly associated with workplace violence training and had a negative association with challenging task training. Furthermore, we demonstrated that self-esteem partially mediated between violence and harassment at work-stress and challenging task training/stress relationships. Finally, we found that stress had a negative relationship with intention to stay.

Our theoretical contribution is the extension of the resource perspective by highlighting the relationship between personal resources and job resources. While it was shown before that personal resources and job resources can play a buffer role between job demands and stress and stress-related outcomes (e.g., burnout), the relationship between personal and job resources required further exploration [4,23]. Our findings contribute to this discussion by empirically demonstrating personal and job resource relationship. Specifically, we show that self-esteem as a personal resource partially mediates the relationship between a job demand (i.e., violence and harassment at work) and stress.

Furthermore, we demonstrate that challenging task training as a job resource has a direct and indirect impact on stress with self-esteem as a mediator. Thus, our model indicates that personal resources can mediate both job demand and stress, and job resource and stress relationships. Furthermore, our findings indicate the elevated importance of personal resources in a high job demand–low job resource context. Future studies can further examine the critical role of personal resources in different contexts testing for other personal resources such as self-efficacy, psychological capital, resilience, and autonomy.

Our findings have important contributions to empirical academic knowledge as well. We found that challenging task training can boost PSWs’ self-esteem and lower their stress directly and indirectly. Furthermore, challenging task training had a positive indirect relationship with intention to stay. This finding is in line with the results from previous studies that demonstrated the positive relationship between training and intention to stay [38]. On the other hand, we showed that workplace violence training did not have any significant associations with self-esteem, stress, or intention to stay. We conclude that challenging task training can help PSWs to alleviate the adverse impact of workplace violence and harassment, whereas workplace violence training might not be as effective. Thus, our study contributes to establishing conflicting findings [7,8] on workplace violence training effectiveness of healthcare workers.

There can be different factors behind the ineffectiveness of workplace violence training. For example, Kelly [10] indicated that PSW training programs, especially those addressing workplace violence, focused more on long-term care settings and not home care settings. It is also important to underline the structural aspects of training effectiveness as well. Regardless of the amount of training the PSWs received, contextual factors such as staff shortages could render the training programs ineffective [10]. Future research can examine the effectiveness of different types of PSW training for prevention and alleviation of violence and harassment at work.

Although this study has several strengths, it is not without limitations. First, our data is cross-sectional. Thus, our findings should not be interpreted as causal. Future studies can use longitudinal or experimental research design to collect data that allows for determining causality. Second, although our province-wide data has a large sample size, it is not possible to know the exact number of PSWs working in Ontario because PSWs do not have a registry in Ontario. Thus, we cannot provide a response rate and the extent to which our findings are representative of PSWs in Ontario and Canada is not clear. Third, we use an encompassing dichotomous conceptualization of violence and harassment experienced by PSWs at work. While this conceptualization allows us to capture an overview of violence and harassment at work, it comes at the expense of examining the intricacies of violence and harassment at work, such as the source and type of violence and harassment. Future research can examine specific aspects of workplace violence and harassment using continuous variables to provide a more nuanced analysis of the impact of job demands, job resources, and personal resources on intention to stay. Finally, this study uses a self-report approach, which may lead to validity issues. That said, while some of our variables, such as our training and violence and harassment–related variables, could be collected at the organizational level for triangulation purposes, other variables, such as self-esteem, stress, and intention to stay, can only be collected following the self-report approach.

## 5. Practice Implications

Our results have important implications for home and community care managers and policy makers. Home and community care organizations operate with a limited budget while facing increased demand from an aging population. Considering the majority of PSWs in our sample are receiving both types of training, knowing the effectiveness of training is necessary for home and community care organizations to maximize their efficiency without going over their budget. Furthermore, it has been shown that effective training can improve retention in organizations, which is an important determinant of efficiency in home and community care organizations [39]. Thus, our findings underline the need for ongoing evaluations of PSW training and redesign of training and intervention programs as needed. In doing so, it is important to include all key stakeholders in discussion. For example, Lipscomb and El Ghaziri [40] argued that healthcare workers should be included in the development and implementation of workplace violence training programs. Furthermore, different training delivery methods should be considered to improve the effectiveness of workplace and violence training provided to PSWs. For example, Gillespie, Farra, and Gates [41] found that a hybrid workplace violence training program designed for healthcare workers that includes online and face-to-face classes was effective in knowledge retention.

In conclusion, this study demonstrates that job resources and personal resources can buffer the detrimental impact of violence and harassment on worker and organizational outcomes in an interactive manner. We developed a model using the resource perspective and tested it with data from PSWs in Ontario, Canada. Our findings shed some light on the resource perspective while underlining the mediating role of self-esteem between training and stress, which might be a direct determinant of PSWs’ intention to stay in their organizations. These results have important implications for home and community care managers and policy makers.

## Figures and Tables

**Table 1:** Means, standard deviations, correlations, and scale reliabilities.

	Variable	Mean	SD	Min	Max	1	2	3	4	5	6	7	8	9	10
1	Intention to stay	11.56	2.76	3	15	*0.91*									
2	Violence and harassment at work (%)	20.91	-	0	1	−0.13 *									
3	Challenging task training (%)	74.09	-	0	1	0.35 *	−0.21 *								
4	Workplace violence training (%)	72.45	-	0	1	0.16 *	−0.031	0.24 *							
5	Self-esteem	24.60	3.72	6	30	0.18 *	−0.11 *	0.19 *	0.04	*0.79*					
6	Stress	28.33	7.81	14	57	−0.26 *	0.23 *	−0.24 *	−0.03	−0.65 *	*0.86*				
7	Tenure (years)	9.80	-	1	38	0.02	0.02	0.00	0.06 *	0.02	−0.02				
8	High school (%)	14.20	-	0	1	0.03	−0.07 *	0.10 *	0.00	0.03	−0.09 *	0.08 *			
9	Trade school (%)	11.64	-	0	1	0.01	0.05 *	−0.11 *	−0.06 *	−0.01	−0.01	−0.01	−0.15 *		
10	College (%)	58.39	-	0	1	−0.02	0.04	−0.01	−0.04	−0.04	0.07 *	0.02	−0.48 *	−0.43 *	
11	University (%)	15.78	-	0	1	−0.02	−0.03	0.02	0.09 *	0.03	−0.00	−0.09 *	−0.17 *	−0.16 *	−0.51 *

Notes: *N* = 1401, * *p* < 0.05, Cronbach’s alpha values are in italics on the diagonal. The mean values of the categorical variables are presented as percentage for ease of interpretation.

## References

[1] Standing Committee on Health (2019) Violence Facing Health Care Workers in Canada, The House of Commons. https://www.google.ca/url?sa=t&rct=j&q=&esrc=s&source=web&cd=1&cad=rja&uact=8&ved=2ahUKEwiq7-zsnvfmAhWjmOAKHeXMCWoQFjAAegQIAxAC&url=https%3A%2F%2Fwww.ourcommons.ca%2FContent%2FCommittee%2F421%2FHESA%2FReports%2FRP10589455%2Fhesarp29%2Fhesarp29-e.pdf&usg=A.

[2] Houshmand M., O’Reilly J., Robinson S., Wolff A. (2012). Escaping bullying: The simultaneous impact of individual and unit-level bullying on turnover intentions. Hum. Relat..

[3] Lanctôt N., Guay S. (2014). The aftermath of workplace violence among healthcare workers: A systematic literature review of the consequences. Aggress. Violent Behav..

[4] Bakker A.B., Demerouti E. (2017). Job demands–resources theory: Taking stock and looking forward. J. Occup. Health Psychol..

[5] Hobfoll S.E. (1989). Conservation of resources: A new attempt at conceptualizing stress. Am. Psychol..

[6] Campbell A.L. (2016). Invisible Workers, Invisible Hazards: An Examination of Psychological and Physical Safety Amongst Workers in Long-Term Residential Care Facilities in the ‘New’ Global Economy.

[7] Anderson C. (2006). Training Efforts to Reduce Reports of Workplace Violence in a Community Health Care Facility. J. Prof. Nurs..

[8] Nachreiner N.M., Gerberich S.G., McGovern P.M., Church T.R., Hansen H.E., Geisser M.S., Ryan A.D. (2005). Impact of training on work-related assault. Res. Nurs. Health.

[9] Keefe J.M., Knight L., Martin-Matthews A., Légaré J. (2011). Key issues in human resource planning for home support workers in Canada. Work.

[10] Kelly C. (2017). Care and violence through the lens of personal support workers. Int. J. Care Caring.

[11] Saari M., Patterson E., Killackey T., Raffaghello J., Rowe A., E Tourangeau A. (2017). Home-based care: Barriers and facilitators to expanded personal support worker roles in Ontario, Canada. Home Health Care Serv. Q..

[12] A Agarwal U., Gupta V. (2018). Relationships between job characteristics, work engagement, conscientiousness and managers’ turnover intentions. Pers. Rev..

[13] Xanthopoulou D., Bakker A.B., Dollard M.F., Demerouti E., Schaufeli W.B., Taris T.W., Schreurs P.J. (2007). When do job demands particularly predict burnout?. J. Manag. Psychol..

[14] Hobfoll S.E. (2011). Conservation of resource caravans and engaged settings. J. Occup. Organ. Psychol..

[15] Westman M., Hobfoll S.E., Chen S., Davidson O.B., Laski S., Perrewe P.L., Ganster D.C. (2004). Organizational stress through the lens of conservation of resources (COR) theory. Research in Occupational Stress and Well Being.

[16] van Woerkom M., Bakker A.B., Nishii L.H. (2016). Accumulative job demands and support for strength use: Fine-tuning the job demands-resources model using conservation of resources theory. J. Appl. Psychol..

[17] Hart P.M., Cooper C.L., Anderson N., Ones D.S., Sinangil H.K., Viswesvaran C. (2001). Occupational Stress: Toward a More Integrated Framework. Handbook of Industrial, Work & Organizational Psychology-Volume 2: Organizational Psychology.

[18] Jex S.M., Elacqua T.C. (1999). Self-esteem as a moderator: A comparison of global and organization-based measures. J. Occup. Organ. Psychol..

[19] Kuster F., Orth U., Meier L.L. (2013). High Self-Esteem Prospectively Predicts Better Work Conditions and Outcomes. Soc. Psychol. Pers. Sci..

[20] Wilkinson C.W. (2001). Violence prevention at work: A business perspective. Am. J. Prev. Med..

[21] Yang H.-C., Ju Y.-H., Lee Y.-C. (2016). Effects of job stress on self-esteem, job satisfaction, and turnover intention. J. Transnatl. Manag..

[22] Xanthopoulou D., Bakker A.B., Demerouti E., Schaufeli W.B. (2009). Reciprocal relationships between job resources, personal resources, and work engagement. J. Vocat. Behav..

[23] Demerouti E., Bakker A.B. (2011). The Job Demands–Resources model: Challenges for future research. SA J. Ind. Psychol..

[24] Albrecht S.L., Marty A. (2017). Personality, self-efficacy and job resources and their associations with employee engagement, affective commitment and turnover intentions. Int. J. Hum. Resour. Manag..

[25] Mobley W.H., Horner S.O., Hollingsworth A.T. (1978). An evaluation of precursors of hospital employee turnover. J. Appl. Psychol..

[26] Steel R.P., Lounsbury J.W. (2009). Turnover process models: Review and synthesis of a conceptual literature. Hum. Resour. Manag. Rev..

[27] Sayin F.K., Denton M., Brookman C., Davies S., Chowhan J., Zeytinoglu I.U. (2019). The role of work intensification in intention to stay: A study of personal support workers in home and community care in Ontario, Canada. Econ. Ind. Democr..

[28] Firth L., Mellor D., Moore K., Loquet C. (2004). How can managers reduce employee intention to quit?. J. Manag. Psychol..

[29] Government of Ontario (2011) Ontario Creating Registry for Personal Support Workers. https://news.ontario.ca/mohltc/en/2011/05/ontario-creating-registry-for-personal-support-workers.html.

[30] Lyons T.F., Cook J.D., Hepworth S.J., Wall T.D., Warr P.B. (1981). Propensity to leave scale of 1971. Experience of Work: A Compendium and Review of 249 Measures and their Use.

[31] Pearlin L.I., Schooler C. (1978). The structure of coping. J. Health Soc. Behav..

[32] Denton M., Zeytinoglu I.U., Davies S., Lian J. (2002). Job Stress and Job Dissatisfaction of Home Care Workers in the Context of Health Care Restructuring. Int. J. Health Serv..

[33] Rodwell J., Demir D. (2012). Oppression and exposure as differentiating predictors of types of workplace violence for nurses. J. Clin. Nurs..

[34] Podsakoff P.M., MacKenzie S.B., Podsakoff N.P. (2012). Sources of method bias in social science research and recommendations on how to control it. Annu. Rev. Psychol..

[35] Podsakoff P.M., MacKenzie S.B., Lee J.-Y., Podsakoff N.P. (2003). Common method biases in behavioral research: A critical review of the literature and recommended remedies. J. Appl. Psychol..

[36] Meyers L.S., Gamst G., Guarino A.J. (2006). Applied Multivariate Research: Design and Interpretation.

[37] Preacher K.J., Hayes A.F. (2008). Asymptotic and Resampling Strategies for Assessing and Comparing Indirect Effects in Multiple Mediator Models. Behav. Res. Methods.

[38] Kim J., Wehbi N., DelliFraine J.L., Brannon D. (2014). The joint relationship between organizational design factors and HR practice factors on direct care workers’ job satisfaction and turnover intent. Health Care Manag. Rev..

[39] Dietz D., Zwick T. (2020). The retention effect of training: Portability, visibility, and credibility^1^. Int. J. Hum. Resour. Manag..

[40] Lipscomb J.A., El Ghaziri M. (2013). Workplace Violence Prevention: Improving Front-Line Health-Care Worker and Patient Safety. N. Solut. A J. Environ. Occup. Health Policy.

[41] Gillespie G.L., Farra S.L., Gates D.M. (2014). A workplace violence educational program: A repeated measures study. Nurse Educ. Pract..

